# Xenbase, the *Xenopus* model organism database; new virtualized system, data types and genomes

**DOI:** 10.1093/nar/gku956

**Published:** 2014-10-13

**Authors:** J. Brad Karpinka, Joshua D. Fortriede, Kevin A. Burns, Christina James-Zorn, Virgilio G. Ponferrada, Jacqueline Lee, Kamran Karimi, Aaron M. Zorn, Peter D. Vize

**Affiliations:** 1 University of Calgary-Computer Science, Calgary, Alberta, Canada; 2 Cincinnati Children's Research Foundation-Division of Developmental Biology, Cincinnati, OH, USA; 3 University of Calgary-Biological Sciences, Calgary, Alberta, Canada

## Abstract

Xenbase (http://www.xenbase.org), the *Xenopus* frog model organism database, integrates a wide variety of data from this biomedical model genus. Two closely related species are represented: the allotetraploid *Xenopus laevis* that is widely used for microinjection and tissue explant-based protocols, and the diploid *Xenopus tropicalis* which is used for genetics and gene targeting. The two species are extremely similar and protocols, reagents and results from each species are often interchangeable. Xenbase imports, indexes, curates and manages data from both species; all of which are mapped via unique IDs and can be queried in either a species-specific or species agnostic manner. All our services have now migrated to a private cloud to achieve better performance and reliability. We have added new content, including providing full support for morpholino reagents, used to inhibit mRNA translation or splicing and binding to regulatory microRNAs. New genomes assembled by the JGI for both species and are displayed in Gbrowse and are also available for searches using BLAST. Researchers can easily navigate from genome content to gene page reports, literature, experimental reagents and many other features using hyperlinks. Xenbase has also greatly expanded image content for figures published in papers describing *Xenopus* research via PubMedCentral.

## INTRODUCTION

Xenbase (http://www.xenbase.org) is the *Xenopus* model organism database. The frogs *Xenopus laevis* and *Xenopus* (*Silurana*) *tropicalis* are widely used in biomedical research, ranging from the study of cell and developmental biology, physiology and drug screening and toxicology. Findings from *Xenopus* research are highly applicable to human health and disease modeling as key biological processes are conserved, such as embryonic germ layer specification, body plan patterning, angiogenesis, neurogenesis and organogenesis. *Xenopus* are amenable to laboratory study as they can be stimulated to lay eggs on demand without seasonal restrictions, and their large robust eggs and embryos serve as powerful *in vivo* systems to explore vertebrate biology. Fertilized embryos develop externally into tadpoles with fully differentiated organs in just a few days when grown in a simple saline solution. Thus, this frog model system is straightforward in observing embryonic development and in detecting changes caused by experimental interventions.

In the 2 years since the last update reported in NAR, the Xenbase team has improved the site's usability by providing a redesigned user interface, adding new content and improving the site's performance and availability. The user interface redesign was done with the help of professional designers, providing Xenbase with a dynamic and easy to navigate landing page. We have added new data types, such as morpholinos and antibodies, as well as increasing the number of our existing data types, such as papers, through manual curation and automatic data harvesting. To improve the site's speed and reduce down time, we replaced Xenbase's hardware with more up-to-date equipment, and completely redesigned the software architecture, allowing us to move to a virtual environment. These content, hardware and software improvements, together with numerous bug and usability fixes, have made the site more useful, stable and responsive.

The scientific method relies heavily on the ability of scientists to reproduce and build upon each other's results. A fundamental principle of reproducibility is clear, unambiguous description of the methods and material resources used in experiments. Consistent use of standardized gene nomenclature, as well as the tools and resources used in *Xenopus* research (e.g. antibodies, morpholinos, transgene (Tg) constructs and transgenic lines) will not only enhance the clarity of the research to the specialist as well as the broader model organism community, but also provide information necessary to facilitate experimental reproducibility. Xenbase continues to support these principles by hosting and promoting the use of stable nomenclature including gene names (congruent with human gene nomenclature), miRNAs, antibodies, morpholinos, transgenic constructs and transgenic frog lines. Our antibody database was launched in early 2013 (1). New antibody entries are continually added, as published, through our manual curation pipeline, giving researchers access to antibody data specifically from work on *Xenopus*. Two newly expanded features on Xenbase further support resources commonly used in *Xenopus* biomedical research: (i) the morpholino module and (ii) transgenic construct and transgenic line nomenclature. These new features are detailed below.

## REDESIGNED LANDING PAGE AND NAVIGATION MAP

**Figure 1.**
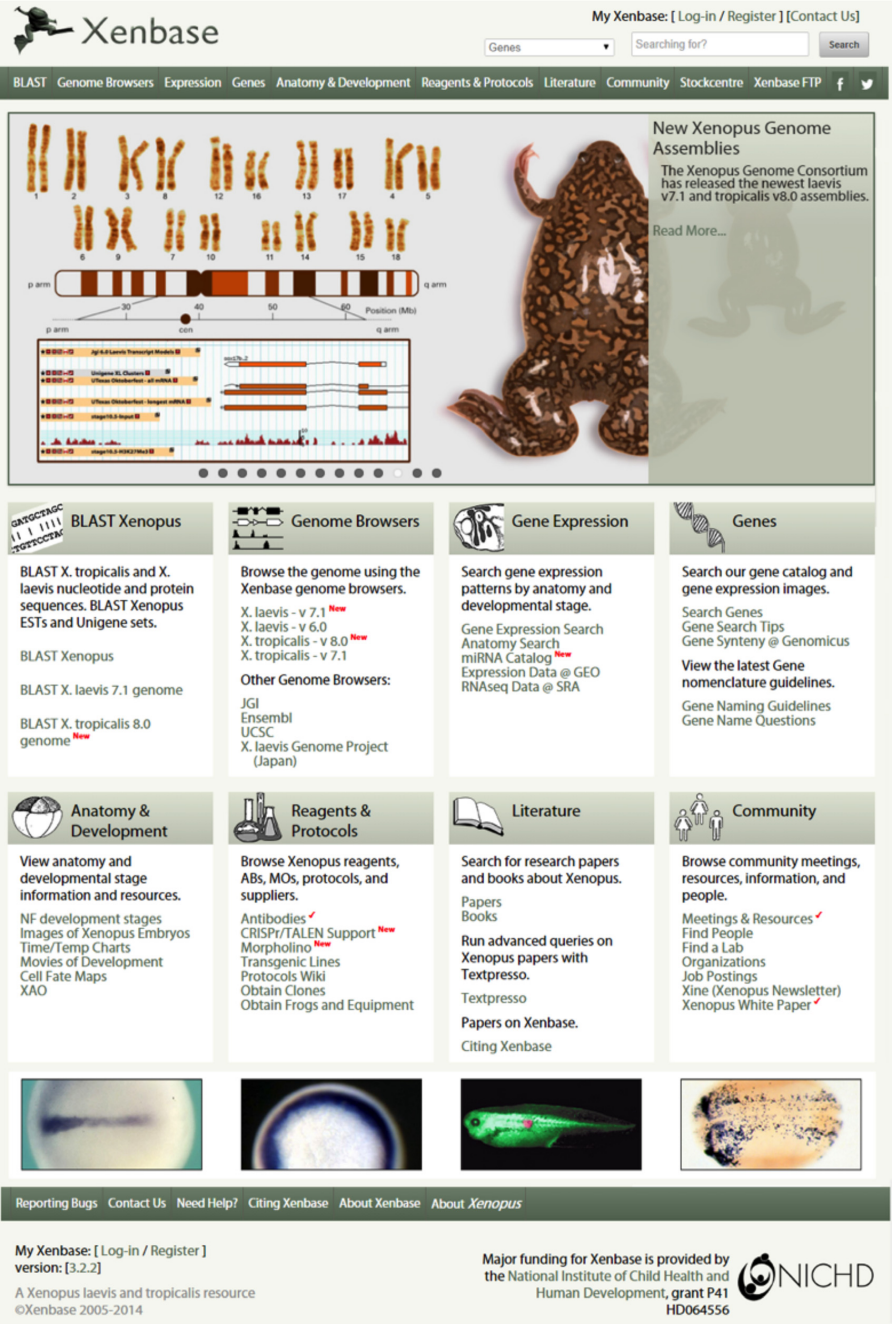
The new Xenbase landing page. The large graphic depicting the *Xenopus* genomes is dynamic, and displays around 10 recent news stories or papers at any one time. Different Xenbase content is organized into tiles, making up the majority of the page. Across the top of the page are login tools, a search tool and a uniform navigation menu found on every Xenbase page. Down the right-hand side are links to many internal and external resources.

In 2013, the Xenbase launched a streamlined interface and reorganized landing page, allowing researchers to better navigate between genome content, gene page reports, Basic Local Alignment Search Tool (BLAST), literature, experimental reagents and many other features (Figure 1). A rotating news slider is now prominently featured on the landing page. The news slider highlights high-profile *Xenopus* research articles, and relevant community information including upcoming conferences, meetings and workshops, scientific award recipients, annotated genome releases, journal special issues and Xenbase updates. Community announcements are further bolstered by a sidebar located on the right side of the landing page. Both the slider and the announcements link to news articles that are stored in a custom MySQL backend database. Archived announcements and news articles are available via hyperlinks. The site's navigation menu was also redesigned. Links in the main body of the landing page have been grouped into tiles with graphical icons. Each tile corresponds to a specific section of the website, such as ‘Gene Expression’, ‘Reagents and Protocols’, ‘Literature’ or ‘Anatomy and Development’. Attention is drawn to new features and update features, indicated by a red check mark. Links to external *Xenopus* resources, as well as other useful resources, such as other Model Organism Databases (MODs), scientific societies and genomic resources, are also located in the sidebar. Xenbase continues to serve our community of *Xenopus* researchers: to date we have 1565 registered researchers, from principal investigators to undergraduate students, and provide information on 181 *Xenopus* research labs. This allows anyone to quickly and easy discover contact information, laboratory websites and key publications from *Xenopus* researchers.

## NEW GENOME BUILDS AND GENE MODELS

The Joint Genome Institute (JGI) submitted new genome builds and gene models to Xenbase, for both *X. laevis* (genome version 7.1, gene model version 7.2) and *X. tropicalis* (genome version 8.0). These genomes and their annotated gene models are available for download from the Xenbase ftp site (ftp://ftp.xenbase.org/pub/Genomics/JGI/). Both genome builds contain chromosome or near chromosome level mappings, and are integrated into the Xenbase database. The genomes can be browsed and searched through GBrowse. Links to gene models are available on individual gene pages, or can be found via BLAST. When BLAST is the selected search method, BLAST results have hyperlinks that take the user directly to the matched model in GBrowse.

## GENOME EDITING SUPPORT

Genome editing technologies using clustered, regularly interspaced, short palindromic repeats/Cas9 endonuclease (CRISPr/Cas) and transcription activator-like nucleases (TALENs) to cause site-directed genome breaks work well in *Xenopus*. Error-prone non-homologous end-joining DNA repair of these cleavages for gene disruption or site-directed gene editing using homologous recombination at the site of the DNA break with co-injection of a donor DNA work well in both *X. tropicalis* and *X. laevis* (2–5). We anticipate these genome editing techniques will be widely used by the *Xenopus* research community and we now provide a link on all gene pages that automatically loads the specific gene sequence and preset parameters directly into the external CRISPr design services, E-CRISP (http://www.e-crisp.org/E-CRISP/designcrispr.html) and E-TALEN (http://www.e-talen.org/E-TALEN/designtalens.html). Xenbase also provides a static page with hyperlinks to a wide range of other CRISPr and TALEN design services and protocol sites (http://www.xenbase.org/other/static/CRISPr.jsp).

## IMPORTATION OF LARGE DATA SETS

An important goal of Xenbase to is archive large data sets that would otherwise be unavailable to the global research community. Often, large-scale screens produce data for many genes for which there is no other gene expression information. These data sets are an incredibly useful resource for researchers looking for novel information. Xenbase's goal is to make these data sets easily accessible. In early 2014, we imported two large data sets from external resources: (i) pronephric kidney expression images from the now defunct EuReGene: The European Renal Genome Project/*Xenopus* Gene Expression Database (XGEbase); (ii) Retinal images from Xenmark/Perron Laboratory/Pollet Laboratory (6). Although these data consist largely of whole mount *in situ* hybridization images, differing metadata and formats required the development of unique scripts to upload each data set.

Xenbase curators are currently collaborating with the Gilchrist Laboratory (NIMR, UK), assessing the results of another large-scale screen for angiogenesis and hematopoietic stem cell specification study from the Patient Laboratory (7,8). Over several years, this work produced over 55 000 gene expression images for 505 genes, covering developmental stages from fertilized egg to tadpole. Images are available for download from our ftp server and digitally selected images will be posted to gene pages and annotated via our standard curation pipeline.

## NEW MIRNA CATALOG

MicroRNAs (miRNAs or miRs) are small, non-coding RNAs that play important roles in regulating gene expression during vertebrate development (9–11). Data and expression profiles of 178 miRNAs in *Xenopus* embryos, generated by the Wheeler lab (University of East Anglia, UK) were uploaded from miRBase (http://www.mirbase.org) and Xenmark (http://genomics.nimr.mrc.ac.uk/apps/XenMARK/). Each miRNA is represented by its official name (e.g. miR-133c (http://www.xenbase.org/geneExpression/static/miRNA/body/xtr-miR-133c.jsp), mature sequence, full sequence when available, one or more expression images and is linked to additional data on miRBase). In total, 693 expression images were imported to the miRNA catalog (http://www.xenbase.org/geneExpression/static/miRNA/body/mirTable.jsp), and is accessible from the Xenbase landing page Gene Expression tile and the Expression drop down menu. A second set of miRNA data was submitted by the JGI and a track displaying predicted miRNAs is available on the *X. laevis* v6.0 GBrowse genome browser. These will be mapped to newer genome builds in the near future. In the long term, Xenbase will develop extensive support for all non-coding RNAs and the miRNA data will migrate into the new module.

## HUMAN DISEASE GENES LINKED TO XENOPUS ORTHOLOGS

*Xenopus* is a particularly powerful system for biomedical research because the developmental and cell biological processes that are discovered in *Xenopus* are highly relevant to mammals, including humans. A long-term goal of Xenbase is to facilitate direct comparison of information from *Xenopus* genes with their human orthologs. Single-gene disease association terms were imported from OMIM (Online Mendelian Inheritance in Man; www.omim.org) and associated with gene pages. Of our 15 845 gene pages, 14 591 have OMIM orthology links, and these are displayed prominently directly under the genes name and function. To find OMIM linked genes using OMIM terms rather than gene names, users can use the search ‘Xenbase with Google’ feature, by entering the OMIM ID number. Search returns will show all gene pages on which the OMIM entry is linked, and all other OMIM disease associations for that gene. For example, entering OMIM ID 31300, for Camurati–Englemann disease, will return the tgfb1 gene page. Clicking on the ‘+’ will expand the display to reveal other known disease associations with tgfb1. Clicking on the disease name will direct researchers to the OMIM entry for that disease. Future development for Xenbase will include adding a specific OMIM term search from the menu bar.

## MORPHOLINO REPRESENTATION

For the past decade, antisense morpholino oligonucleotides (MOs) have been extensively used in *Xenopus* to suppress gene function (12,13). In order to further support *Xenopus* researchers to quickly identify relevant and effective reagents for their research, we have constructed an integrated database module to catalog published MO reagents. This new morpholino feature currently contains information on over 1600 published MO reagents, with ∼700 coming from manual literature curation, and the remaining 900 via the literature using the Xenbase implementation of the automatic text mining tool, Textpresso (14). By querying ‘morpholino’ or ‘MO’, the Textpresso tool allowed us to quickly find MO sequences and references that were in the same paragraph as the query text, thus greatly reducing the amount of time it took to record the information from roughly 1300 published articles with keyword ‘morpholino’. Unique MO names (using the basic format of ‘gene symbol MO#’ where sequential numbers are given in order of curation) and database identifiers (XB-MORPHOLINO-####) were assigned to each published MO. Reagent names used in the literature are recorded as synonyms. Each MO is attributed to the published literature in which they are used, helping *Xenopus* researchers find additional information, such as concentration used, microinjection location, resulting phenotypes and rescue experiments. A publicly accessible wiki page is available on each MO page so that the community can add and view additional information on any individual MO.

**Figure 2.**
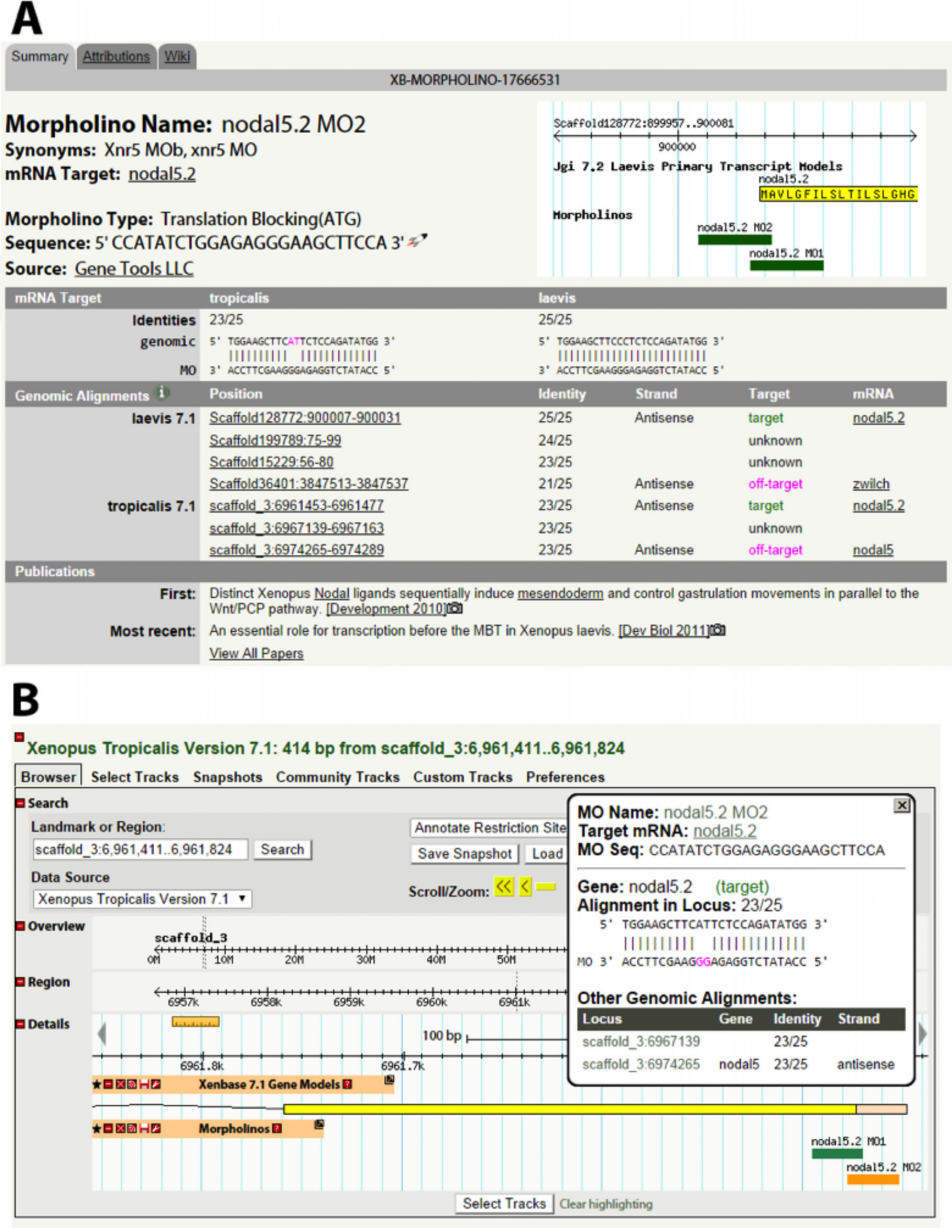
The new morpholino support interface. We store data on over 1000 morpholino reagents, gathered from large-scale screens and the *Xenopus* literature. (A) Each morpholino sequence is aligned to the genome via BLAST and the results for both on-target and potential off-target matches displayed. Various other data, such as publications, utilizing the reagent are also illustrated. (B) Morpholino sequences are also available as a track in our GBrowse implementation. When a user clicks on a morpholino in the browser details on the alignment are displayed in a pop-up window.

The MO interface displays (i) name and synonyms; (ii) mRNA target; (iii) MO type (translation blocking, splice blocking); (iv) sequence; (v) mRNA target alignments and identities; (vi) genomic alignments (including position, identity, mRNA name, on-target/off-target designation and sense/antisense designation) and (vii) attributed publications (Figure 2). The morpholino feature can be accessed at the url, http://www.xenbase.org/reagents/morpholino.do and under the ‘Reagents & Protocols’ drop down menu.

Most morpholinos are ∼25 nt long, which allows them to be designed against unique sequences within the genome. However, morpholino-to-RNA-binding may still occur if one or several of the nucleotides are mismatched. This flexibility can be beneficial in that researchers may design mopholinos that target all closely related paralogues of a gene family with a single MO, or design a single morpholino that works for the *X. tropicalis* gene, as well as the two *X. laevis* homologs. However, morpholinos may also bind unintended off-target sequences elsewhere in the transcriptome. The resulting phenotype may be a result of off-target gene knock-downs. To help users avoid non-specific phenotypes, all potential genomic alignments, including off-target alignments, are displayed. To do so, we perform an automated analysis that checks for (i) a minimum genomic sequence similarity, (ii) presence of a gene model at a given locus, (iii) whether the gene model is the intended target of the MO and (iv) whether the morpholino would hybridize to the intended biologically relevant sense strand target or the irrelevant antisense strand.

Each antisense MO sequence is aligned against the *X. tropicalis* (build 7.1) and *X. laevis* (build 7.1) genomes using BLAST, and all alignments with up to four mismatches are listed for the user. These alignments are displayed with the correlating scaffold/chromosome position, strand and mRNA target with gene models. If the alignment was within a gene model, the alignment is labeled with the gene name. This gene name is compared to the manually curated mRNA target to assign ‘on-target’ or ‘off-target’ labels. The ‘on-target’ designation is independent of species and subgenome (e.g. an alignment for a cxcr4 morpholino is ‘on-target’ if it aligns to the *X. tropicalis* cxcr4 gene, or the *X. laevis* cxcr4.L or cxcr4.S homologs). Finally, the alignment is compared to the mRNA strand of the locus gene to determine if the specific alignment is sense or antisense (Figure 2).

All alignments from each morphlino in the database are exported to a GBrowse GFF3 track next to the gene model track allowing quick reference to the location of the morpholino target within the gene model. The MO bands are color-coded within GBrowse reflecting the quality of their alignment: green is 0–1 mismatch, orange is 2–3 mismatches and red is 4 mismatches. By clicking a MO alignment, the MO name, target and sequence can be seen in a pop-up window. The pop-up also shows information for the local MO alignment, including the gene name (if there is a current gene model), the alignment being ‘on-target/off-target’, the nucleotide identity, and whether the target of the morpholino is transcribed. Furthermore, there are links to other genomic hits for the morpholino, along with genes at other loci, alignment identities and whether those alignments are sense/antisense (Figure 2B).

## EXPANDED GENE NOMENCLATURE GUIDELINES

*Xenopus* genes and Tg are named in accordance to the guidelines established by the *Xenopus* gene nomenclature committee, a group of senior *Xenopus* researchers. The gene nomenclature guidelines were based on human gene nomenclature guidelines. *Xenopus*-specific additions were added; for instance, the designation of ‘L’ and ‘S’ genes from the *X. laevis* subgenomes. The ‘Gene Nomenclature Guidelines for *Xenopus*’ are posted on the Xenbase website (http://www.xenbase.org/gene/static/geneNomenclature.jsp).

In consultation with the *Xenopus* stock centers in the United States (the National *Xenopus* Resource, NXR), in the United Kingdom (the European *Xenopus* Resource Center, EXRC) and in Japan (the National BioResource Project, NBRP), Xenbase has drafted the ‘*Xenopus* Transgenic Nomenclature Guidelines’. These guidelines cover the naming of transgenic constructs, mutant and transgenic *Xenopus* lines and are based on the well-established transgenic naming rules for zebrafish (http://zfin.org/) and mouse (http://www.informatics.jax.org/). As in other models, the name of a transgenic line is based on name of the transgenic construct(s) used to generate the line. As both *X. laevis* and *X. tropicalis* transgenic lines are routinely established, sometimes using the same Tg construct, identifying species in the official name of each Tg line was deemed necessary. Thus, the letters Xl for *X. laevis*, or Xt for *X. tropicalis*, will precede the Tg line official name. The laboratory of origin of each Tg line will also be indicated using uniques laboratory codes, as registered with the ILAR (http://dels.nas.edu/ilar/). The basic format for Tg construct names will be italicized, *Tg(promoter:ORF/gene)*, for example, *Tg(mlc2:GFP)*, where the gene mlc2 (myosin), light chain 2, regulatory, cardiac, slow drives GFP expression in the cardiac mesoderm. An example of a *X. laevis* line established using this construct would be named Xl.Tg(mlc2:GFP)^1Mohun^ where the laboratory code (in this example the Mohun Laboratory), preceded by a line serial number, is added in superscript, and the whole name of the line is in standard script (not italics, as is the construct). Importantly, this stable Tg line will always carry this official name, and any legacy names will be recorded as synonyms, which will be searchable. Diverging somewhat from the other models, in *Xenopus*, Tg names are purposefully designed to be less cumbersome and confusing, and will omit some technical details from the construct name (such as the length of insertions). Instead, we record and display complete details in the construct or line description on Xenbase. The detailed transgenic naming guidelines are posted on Xenbase (http://www.xenbase.org/gene/static/tgNomenclature.jsp).

Authors are strongly encouraged to follow the guidelines when naming genes, Tg constructs and Tg *Xenopus* lines. Xenbase can assist with queries, help to apply the guidelines consistently and liaise with the HGNC and/or the *Xenopus* nomenclature committee to resolve any naming issues for genes and transgenics. Xenbase is currently helping the *Xenopus* stock centers to rename all currently available Tg lines, and is redesigning a more streamlined interface for Tg curation.

## XENOPUS SCIENTIFIC LITERATURE-EXPANDED FIGURE COVERAGE VIA PubMedCentral (PMC)

In the literature section of Xenbase, we display information on papers in PubMed using ‘*Xenopus*’ in the title or abstract. Xenbase now hosts a compendium of ∼45 700 research articles and we have brokered image reproduction agreements with 20 research journals. In addition to figures from these journals, we display information from ∼70 open access journals which regularly publish *Xenopus* research, several of which we post display figures from papers 2–6 months post-publication. Curators use Java-based custom screen-scrapers (14) to select specific figures from journal websites. These figures are imported with their associated figure legend text using both automated and manual annotation (15). As many publishers now make much of their content publicly available through PMC, and many of the figures are covered by Open Access Subset, we performed a batch import of all figures associated with *Xenopus* papers already represented in Xenbase available from this resource. This import added 13 538 figures linked to 1956 papers, greatly improving the value of our literature content. To date, Xenbase displays 4323 *Xenopus* research papers with figures containing gene expression images. This represents ∼10% of all *Xenopus* literature in our database.

A number of text-mining scripts identify keywords in abstracts and figure legends, including all gene names, gene symbols and synonyms, and tissues or anatomy terms represented in the *Xenopus* Anatomical Ontology (XAO)(16). Researchers can directly go to the gene page or the XAO term page, respectively, using hyperlinks. Additional new link types have been added to literature records, including links to morpholino pages when a paper contains morpholino data, links to gene pages when it contains data pertinent to a specific gene and links to antibody pages when the paper has used specific antibodies. Unique PMC identifiers were now added to each article, and relevant copyright notices are affixed to each downloaded image. For details on the Open Access Subset please see: http://www.ncbi.nlm.nih.gov/pmc/tools/openftlist/.

Full-text versions of all papers are downloaded whenever our host university has a digital subscription. These full-text versions are not made available to the public for copyright reasons, but are accessible to the Textpresso text-mining system. This allows researchers to perform powerful combinatorial queries of the *Xenopus* literature. Textpresso queries are returned to the user as marked-up text highlighting identified passages in publications and a link to allow the user to jump to the full paper if their IP address has appropriate access permissions.

Xenbase is now linked from external sites including Elsevier's ScienceDirect and NCBI/PubMed's LinkOut. These new links allows readers to jump from these external resources directly to relevant Xenbase content. Xenbase is open to supporting other such initiatives as opportunities arise.

## XAO DEVELOPMENT

The XAO is a controlled vocabulary of terms that represent the anatomy and development of *Xenopus* from unfertilized oocyte to the adult frog. The XAO also maps the lineages of tissues and the timing of each developmental stage. The XAO underlies Xenbase's manual annotation of temporal and tissue-specific gene expression. Our pipeline for XAO development has been described previously (16). The previous version of the XAO prevented us from capturing tissue-specific gene expression at the detailed level presented in a body of *Xenopus* neuroanatomy literature spanning several decades. Our most recent revision focused on adding anatomy terms to better capture the subregionalization of the central nervous system. To achieve this, we added terms such as ‘basal ganglia’ to capture larger subregions, and we added terms such as ‘caudal amygdala’ to represent individual nuclei. An emphasis was placed on structures with analogous structures in humans; structures that commonly demonstrate developmental tissue-specific gene expression, which in turn correspond to biologically important differences in the mature structures, as in where neurotransmitters are expressed. We selected the most common terms from authoritative papers in the recent neurobiology literature (17–19). Another field of expanding basic research using *Xenopus* is the study of ciliopathies and mucociliary disease (20–23). To accurately capture gene expression in this field, we added several terms to the XAO supporting curation of expression of genes in ciliated versus non-ciliated epithelia cell types, and substructures of epithelial tissues. In total, 89 new terms with complete definitions and relationships terms where added to the XAO v3.0. Five XAO tracker issues were resolved through SourceForge and Google Code, in addition, 134 new synonyms, 87 new *part_of* relationships and 17 new *develops_from* relationships were added to improve the comprehensiveness of the XAO. XAO v3.0 is available in OBO and OWL (Web Ontology Language) formats, and can be downloaded from the Xenbase FTP site (ftp://ftp.xenbase.org) and from OBO Foundry (http://www.obofoundry.org). Researchers can browse the XAO for terms, term definitions and developmental stages under the Anatomy and Development section on Xenbase. We encourage researchers to send term requests, comments and synonym recommendations via the XAO tracker (http://sourceforge.net/p/obo/xenopus-anatomy-xao-term-requests/).

## VIRTUALIZATION AND THE MOVE TO THE CLOUD

All Xenbase systems used to run on a single web server. In mid-2013, Xenbase moved to a private cloud environment to improve stability and performance. We separated the main software components, such as the application server, the database, the FTP server and the BLAST+ server, and moved them to separate Virtual Machines (VMs). Having the main components run in separate environments has eased the process of management and upgrading, as each VM can be brought offline or updated without affecting the other components. All VMs run on a pair of identical physical servers running VMware 5.1, and communicate with each other through a dedicated network (if running on different physical servers), or though shared memory (if running on the same server). Having two physical servers provides redundancy and hardware fault tolerance. VMs can automatically migrate between the two physical VMware servers to provide optimum performance, load balancing and fault tolerance. This multiserver system provides excellent performance, and VM restarts take a fraction of the time required to reboot as the previous monolithic server. We also have a number of VMs for a separate development and testing environments, each closely matching the production environment, and allowing us to develop new code and resolve bugs with minimal interference to the production site. Details of virtualization will be fully described elsewhere (Karimi and Vize, Database; in press).

## CITING XENBASE

If Xenbase content or services contribute significantly to a publication or to research success, researchers are suggested to cite this article. Further reference specific to the gene expression, database and the XAO can been found here: http://www.xenbase.org/other/static/citingXenbase.jsp.
